# Efficacy and safety of six immunoadsorption treatments for severe lupus nephritis: a Bayesian network meta-analysis and systematic review

**DOI:** 10.3389/fimmu.2026.1661291

**Published:** 2026-03-13

**Authors:** Jiazhen Liu, Yin Zheng, Xuxin Zhang, Jiani Xia, Kewei Jia, Yi Zhao

**Affiliations:** 1 Liaoning University of Traditional Chinese Medicine, Shenyang, Liaoning, China; 2 Department of Chinese Medicine, The First Hospital of China Medical University, Liaoning, China

**Keywords:** Bayesian model, DNA280 adsorption column, HA280 adsorption column, PH-350 adsorption column, staphylococcal protein A

## Abstract

**Background:**

Severe lupus nephritis (LN) remains difficult to manage despite standard immunosuppressive therapy. Immunoadsorption (IA) has been increasingly used as an adjunctive treatment. However, comparative evidence across different IA columns is limited.

**Methods:**

We conducted a systematic review and Bayesian network meta-analysis of randomized controlled trials evaluating six IA columns for severe LN. PubMed, Embase, Scopus, Web of Science, VIP, Wanfang, and CNKI were searched up to January 2025. Outcomes included disease activity (Systemic Lupus Erythematosus Disease Activity Index, SLEDAI), renal parameters, immunological markers, and adverse events. Risk of bias was assessed using the Cochrane risk-of-bias tool, and Bayesian network meta-analysis was performed in R (version 4.4.1).

**Results:**

A total of 24 randomized controlled trials involving 1,442 patients were included. DNA280 plus conventional pharmacotherapy showed a favorable probability ranking for SLEDAI improvement. DNA280 plus plasma exchange (PE) plus conventional pharmacotherapy showed a statistically significant advantage *versus* conventional pharmacotherapy alone [mean difference (MD) = 1.8, 95% credible interval (95%CrI) = 0.05–3.5] for 24-h proteinuria and ranked favorably across several renal outcomes. PH-350 plus conventional pharmacotherapy showed a favorable probability ranking for serum creatinine improvement. Adverse events were reported in 17 studies; however, comparative safety analyses were not feasible due to inconsistent definitions and reporting.

**Conclusions:**

IA strategies—in particular DNA280-based regimens—may offer relative advantages for severe LN. However, the evidence is limited by study quality, heterogeneity, sparse comparisons for some columns, and reliance on surrogate outcomes. The findings should be interpreted as hypothesis-generating, and higher-quality comparative trials with clinically meaningful endpoints are needed.

**Systematic Review Registration:**

https://www.crd.york.ac.uk/PROSPERO/, identifier CRD420251031348.

## 1 Introduction

Systemic lupus erythematosus (SLE) is a multisystem autoimmune disease, among which lupus nephritis (LN), one of its most severe complications, affects approximately 40%–60% of patients with SLE (1). Severe LN, as the most critical target organ damage in SLE, is classically characterized by the clinical triad of 24-h proteinuria ≥3.5 g, serum creatinine (Scr) >1.5 mg/dl, and active urinary sediment. Its pathological features manifest as International Society of Nephrology/Renal Pathology Society (ISN/RPS) class IV or class V + IV lesions, often accompanied by persistent hypocomplementemia and high-titer anti-dsDNA antibodies (2). Without timely and effective intervention, patients rapidly progress to end-stage renal disease (ESRD), with significantly elevated risks of cardiovascular events and infection-related mortality (3, 4). Studies indicate that despite continuous advances in therapeutic approaches, approximately 25% of patients with LN still progress to ESRD. The incidence of LN is significantly higher in Asian populations than in European and American populations, suggesting that genetic and environmental factors may drive heterogeneity in renal damage (5, 6). The current standard treatment primarily involves glucocorticoids combined with cyclophosphamide or mycophenolate mofetil. However, treatment resistance or cumulative drug toxicity may lead to secondary injury (7). Thus, there is an urgent clinical need for more precise immunomodulatory strategies.

Immunoadsorption (IA) is a novel technique leveraging specific antigen–antibody binding. It selectively removes pathogenic autoantibodies and immune complexes through highly specific adsorption and reduces complement activation products and inflammatory mediator release, thereby controlling the disease activity and preserving organ function (8). IA therapy can block the immune-inflammatory cascade driving renal injury. Unlike plasma exchange (PE), IA preserves beneficial proteins, reducing complications such as coagulopathy or hypoalbuminemia, demonstrating unique advantages in the treatment of severe LN. By reviewing the relevant literature and analyzing data, we identified six IA columns most commonly used in clinical LN treatment: staphylococcal protein A, HA280, DNA280, PH-350, Ig-Therasorb, and GAM146. Current clinical studies on IA columns for LN predominantly involve pairwise comparisons, lacking data on the efficacy differences among multiple columns, which limits applicability in complex clinical scenarios. Network meta-analysis addresses this through the construction of multi-arm comparison models, enabling direct or indirect cross-trial comparisons of efficacy across different adsorption columns. This provides evidence-based guidance for clinicians for the selection of optimal IA regimens in the absence of direct comparative evidence.

## 2 Materials and methods

### 2.1 Database and literature search strategy

We performed computerized searches in the PubMed, Embase, Scopus, Web of Science, VIP, Wanfang, and CNKI databases for randomized controlled trials (RCTs) relevant to this study. The search time frame extended up to January 2025. Key search terms included: lupus nephritis, systemic lupus erythematosus, immunoadsorption column, staphylococcal protein A adsorption column, HA280 adsorption column, DNA280 adsorption column, *PH-350 adsorption column*, Ig-Therasorb column, GAM146 adsorption column, and immunoadsorption therapy. **Supplementary Table 1** shows the details of the comprehensive search strategy.

### 2.2 Inclusion criteria

The inclusion criteria were as follows:

a) Study type: RCTs published before January 2025 comparing different IA columns or IA columns *versus* control treatments for LN, irrespective of blinding. The design, conduct, and reporting of this study adhered to the Preferred Reporting Items for Systematic Reviews and Meta-Analyses for Network Meta-Analyses (PRISMA-NMA) guidelines.
b) Study population: Patients definitively diagnosed with LN meeting the 1997 revised American Rheumatism Association (ARA) classification criteria for SLE (8) or the 2003 ISN/RPS classification criteria (9). All patients had confirmed renal involvement by renal biopsy, with pathological types being class IV or class V + IV, confirming severe LN cases; no allergy or intolerance to IA; absence of severe comorbidities (e.g., congestive heart failure or life-threatening pulmonary dysfunction); no severe bacterial or active viral infections (e.g., hepatitis B or hepatitis C); no coagulation abnormalities, bleeding tendency, or active bleeding; and no restrictions on age or sex.
c) Interventions: Comparisons between different IA therapies combined with conventional pharmacotherapy (glucocorticoids combined with either cyclophosphamide or mycophenolate mofetil) or comparisons between IA combined with conventional pharmacotherapy and conventional pharmacotherapy alone.
d) Outcome measures: 1) Systemic Lupus Erythematosus Disease Activity Index (SLEDAI) score; 2) 24-h proteinuria; 3) blood urea nitrogen (BUN); 4) anti-dsDNA antibodies; 5) Scr; and 6) adverse events.

### 2.3 Exclusion criteria

We excluded: a) studies not published in Chinese or English; b) study populations not definitively diagnosed with LN meeting the inclusion criteria; c) those with missing, erroneous, or unobtainable key outcome data (even after contacting the authors); d) duplicate publications; e) experience summaries, case reports, animal experiments, and reviews; and f) studies that failed to specify the IA column used.

### 2.4 Literature screening and data extraction

Literature retrieved from the databases was initially deduplicated and screened using EndNote software. After integrating the search results, the full texts were downloaded for further screening to exclude studies that did not meet the inclusion criteria. For missing data or other critical information in the literature, attempts were made to contact the original authors. Two researchers independently performed these tasks and extracted data using a pre-designed form. Discrepancies were resolved through consultation with a third researcher. Non-English literature was independently translated by bilingual researchers with medical backgrounds, and accuracy of the key terminologies was verified by rheumatology/immunology experts. Ultimately, 24 studies were included. Data were extracted into an Excel spreadsheet, including: the first author, publication year, country, journal, sample size (men/women), age, interventions, treatment duration, and outcome measures.

### 2.5 Risk of bias assessment

The methodological quality of the included studies was assessed using the Cochrane risk-of-bias tool. Risk of bias was evaluated across the following domains: random sequence generation, allocation concealment, blinding of participants and personnel, blinding of outcome assessment, incomplete outcome data, selective reporting, and other sources of bias. The assessment was performed independently by two reviewers, with disagreements resolved by discussion or consultation with a third reviewer. Risk-of-bias figures were generated using Review Manager (RevMan) 5.4.

### 2.6 Statistical analysis

The Bayesian network meta-analysis was conducted under the assumptions of transitivity and consistency. Continuous outcomes were summarized as mean differences (MDs) with 95% credible intervals (CrIs), and dichotomous outcomes (when available) were assessed as odds ratios (ORs) or risk ratios (RRs) with 95%CrIs. A comparison was considered statistically significant when the 95%CrI excluded 0 for continuous outcomes or 1 for ratio measures.

The network meta-analysis was implemented in R (e.g., the gemtc package) using Markov chain Monte Carlo methods. Four Markov chains with overdispersed initial values were run with a burn-in of 5,000 iterations followed by 20,000 sampling iterations; if convergence was not satisfactory, the iterations were extended to 50,000. Convergence was assessed using the potential scale reduction factor (PSRF; now referred to as R-hat), with values close to 1.0 indicating adequate convergence. When closed loops were present in the evidence network, inconsistency was examined using the node-splitting approach (10). Rank probability plots were also generated to describe the probability that each intervention achieved each possible rank; however, the ranking probabilities were interpreted as relative comparative tendencies rather than evidence of superiority, unless supported by corresponding CrIs (11). Between-study heterogeneity was assessed using the *I*^2^ statistic, with *I*^2^ ≥ 50% indicating substantial heterogeneity. Small-study effects were explored using comparison-adjusted funnel plots and Egger’s test, noting that power may be limited when the number of studies is small. To assess the robustness of the primary findings, sensitivity analyses were conducted by rerunning the network meta-analysis under a fixed-effects model, and the treatment rankings were compared with those obtained from the random-effects model.

## 3 Results

### 3.1 Literature search results

**Figure 1:**
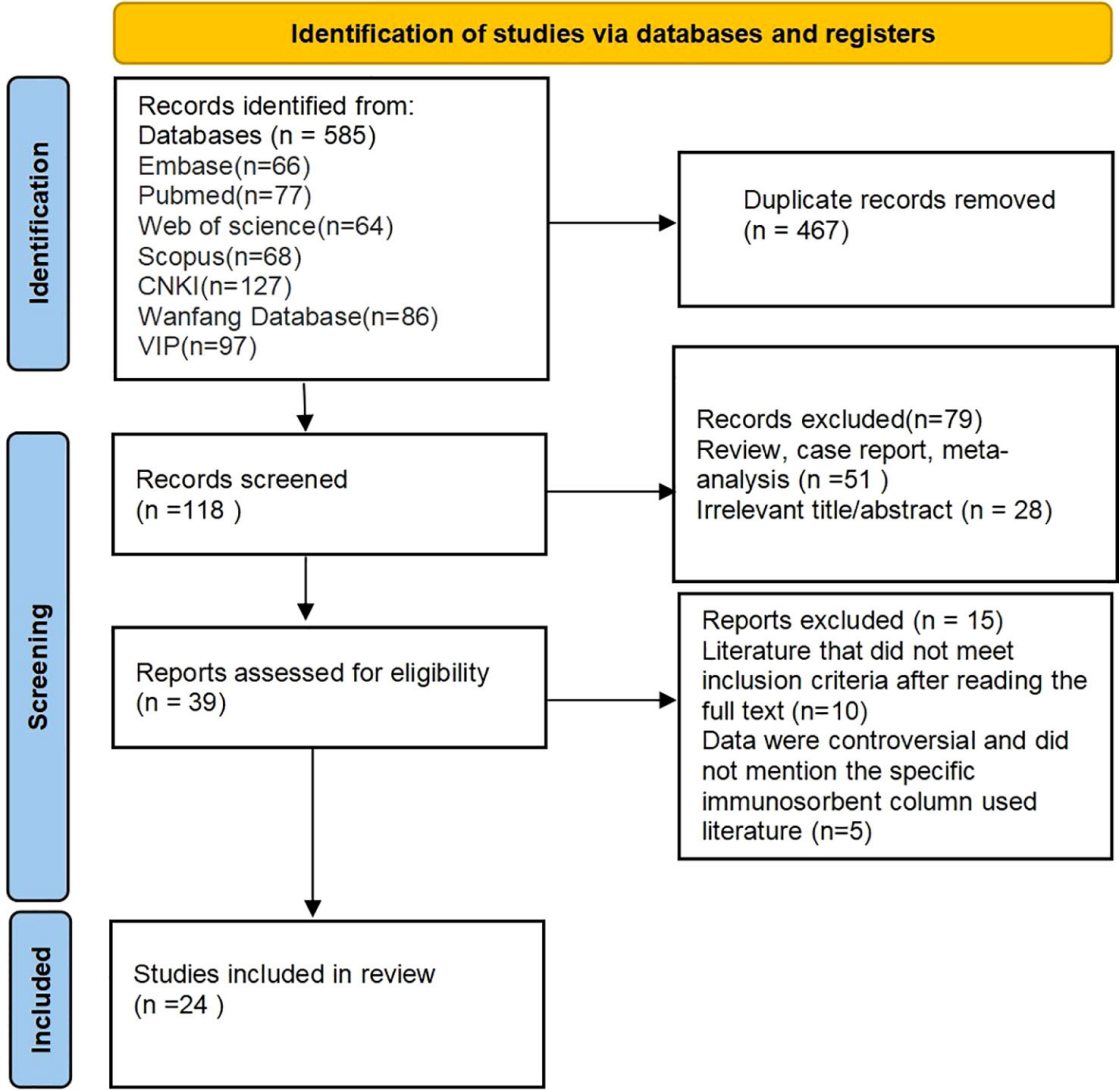
PRISMA flowchart.

The initial search yielded 585 articles. All records were imported into EndNote software for deduplication, resulting in the removal of 467 duplicate articles. After reviewing the titles and abstracts, 79 articles were excluded. Full texts were then screened, leading to the exclusion of 10 articles that did not meet the inclusion criteria, along with five articles that were disputed or failed to specify the IA column used. Ultimately, 24 articles were included (12–35), comprising five English-language articles (12–16) and 19 Chinese-language articles (17–35). The literature screening flow is presented in **Figure 1**.

### 3.2 Basic characteristics of the included studies and results of the risk of bias assessment

**Figure 2:**
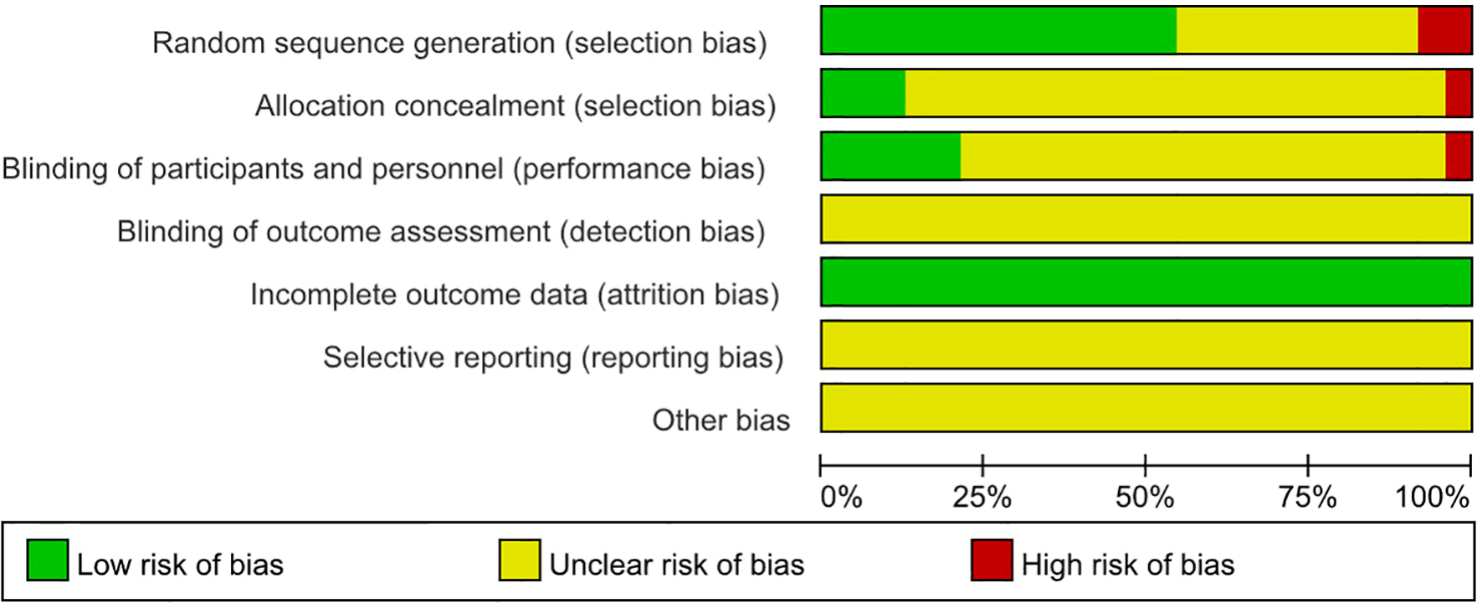
Risk of bias assessment proportion plot.

The 24 included studies (12–35) encompassed a total sample size of 1,442 patients. Of these, 12 studies reported the SLEDAI scores (12–15, 18–20, 22, 24, 27, 28, 31), 14 studies reported 24-h proteinuria (13, 16, 19, 20, 22–24, 27–30, 32, 34, 35), and 10 studies reported BUN (19, 23, 24, 26, 27, 29, 30, 32, 33, 35). There were 13 studies that reported anti-dsDNA antibodies (14–17, 19, 22, 24, 25, 28–31, 33), and 12 studies reported Scr (19, 20, 23, 24, 26, 27, 29, 30, 32–35). Regarding risk of bias, 13 studies described specific randomization methods (12, 14, 16, 17, 20, 22–24, 26, 29–31, 34), three studies used sealed opaque envelopes for allocation concealment (20, 22, 34), and five studies employed a single-blind design (participants blinded) (17, 20, 24, 29, 30). None of the included studies described blinding of the outcome assessors; therefore, this domain was judged as unclear risk. The risk of bias due to incomplete outcome data was low. Risks of other biases and selective reporting were unclear. The basic characteristics of the included studies are presented in **Table 1**, and the risk of bias assessment is shown in **Figure 2**.

**Table 1:** Characteristics of the included studies.

Author (year)	Country	*N* (M/F)	Average age (years)	Intervention (T/C)	Treatment (weeks)	Outcome^a^
Chee-Yean Loo 2010 (12)	Malaysia	T: 0/14 C: 4/10	T: 30.2 ± 7.5 C: 31.9 ± 11.6	PH-350 + GC, CTX/PE + GC, CTX	4	1 and 6
Biesenbach 2009 (13)	Austria	T: 0/6 C: 0/2	T: 32.5 ± 10.9 C: 38.5 ± 3.5	Protein A columns + GC, CTX/Gam146 peptide columns + GC, CTX	12	1, 2, 4, and 6
Gaubitz 1998 (14)	Germany	T: 3/7 C: 1/9	T: 40.7 ± 12.0 C: 31.0 ± 12.0	PH-350 + GC, CTX/Ig-Therasorb + GC, CTX	6	1, 4, and 6
Stummvoll 2004 (15)	Austria	2/7 3/22 1/6 1/14	29.0 ± 11.6 31.0 ± 9.4 30.1 ± 7.9 36.3 ± 13.9	Ig-Therasorb + GC, CTX/CTX/Ig-Therasorb/Gam146 peptide columns + GC, CTX	12	1, 4, and 6
Xu 2015 (16)	China	T: 0/31 C: 0/26	T: 32.28 ± 14.45 C: 34.05 ± 11.53	HA280 + GC, CTX/DNA280 + GC, CTX	12	2, 4, and 6
Liang 2017 (17)	China	–	–	DNA280 + GC, CTX/GC, CTX	4	4 and 6
Chen 2011 (18)	China	T: 3/27 C: 4/26	T: 34 ± 17 C: 32 ± 15	DNA280 + GC, MMF/GC, MMF	3	1 and 6
Xiao 2015 (19)	China	T: 2/6 C: 2/10	T: 33 ± 6 C: 32 ± 10	DNA280 + GC, CTX/GC, CTX	12	1–6
He 2016 (20)	China	T: 3/22 C: 4/21	T: 27.1 ± 2.6 C: 27.0 ± 2.5	DNA280 + GC, HCQ/GC, HCQ	3	1, 2, and 5
Xie 2024 (21)	China	T: 1/15 C: 2/14	T: 40.13 ± 10.54 C: 45.56 ± 14.89	HA280 + GC, CTX/GC, CTX	24	1, 2, 5, and 6
Kong 2013 (22)	China	T: 4/47 C: 5/50	T: 32.51 ± 11.39 C: 33.67 ± 11.72	PH-350 + GC, CTX/GC, CTX	24	1, 2, 4, and 6
Yang 2019 (23)	China	T: 4/26 C: 3/27	T: 36.2 ± 2.3 C: 36.2 ± 2.3	Protein A columns + GC, CTX/DNA280 + GC, CTX	8	2, 3, and 5
Xuan 2014 (24)	China	T: 2/39 C: 4/37	T: 34.2 ± 9.8 C: 36.5 ± 10.2	DNA280 + GC, MMF/GC, MMF	4	1–6
Yang 2015 (25)	China	T: 14/26 C: 16/28	T: 26.0 ± 5.2 C: 26.2 ± 5.3	DNA280 + GC, CTX/GC, CTX	24	4 and 6
Yao 2002 (26)	China	T: 0/15 C: 0/15	T: 31.2 ± 12.1 C: 33.4 ± 12.7	Protein A column + GC, CTX/GC, CTX	4	3, 5, and 6
Han 2008 (27)	China	T: 8/44 C: 3/27	T: 38.2 ± 14.5 C: 33.5 ± 12.6	Protein A column + GC, CTX/GC, CTX	24	1, 2, 3, and 5
Kong 2011 (28)	China	T: 7/32 C: 7/32	T: 32.31 ± 11.49 C: 34.87 ± 11.92	PH-350 + GC, CTX/GC, CTX	24	1, 2, 4, and 6
Wang 2019 (29)	China	T: 19/22 C: 18/23	T: 32.54 ± 13.26 C: 30.25 ± 11.17	DNA280 + PE + GC, CTX/DNA280 + GC, CTX	4	2–6
Long 2018 (30)	China	T: 10/14 C: 8/16	T: 47.61 ± 5.22 C: 57.51 ± 4.62	DNA280 + GC, CTX/GC, CTX	12	2–5
Gao 2017 (31)	China	T: 5/42 C: 3/44	T: 35.3 ± 7.4 C: 36.5 ± 5.5	PH-350 + GC, CTX/GC, CTX	4	1 and 4
Li 2009 (32)	China	T: 2/15 C: 3/16	T: 27 ± 15.3 C: 27 ± 15.6	DNA280 + GC, CTX/GC, CTX	24	2, 3, 5, and 6
Lai 1999 (33)	China	T: 3/17 C: 2/20	T: 30.8 ± 11.6 C: 32.4 ± 13.7	PH-350 + GC, CTX/GC, CTX	4	3–6
Zhu 2017 (34)	China	T: 2/23 C: 4/21	T: 38.98 ± 8.93 C: 39.12 ± 9.56	DNA280 +GC/GC	3	2 and 5
Li 2005 (35)	China	–	T: 38.5 C: 38.5	DNA280 + PE + GC, CTX/PE + GC, CTX	4	2, 3, and 5

An en dash indicates that the intervention was excluded from the metric.

*N*, sample size; *M*, male; *F*, female; *T*, trial group; *C*, control group; *GC*, glucocorticoids; *CTX*, cyclophosphamide; *MMF*, mycophenolate mofetil; *HCQ*, hydroxychloroquine; *PE*, plasma exchange.

a The following outcomes were assessed: 1) Systemic Lupus Erythematosus Disease Activity Index (SLEDAI) score; 2) 24-h proteinuria; 3) blood urea nitrogen (BUN); 4) anti-dsDNA antibodies; 5) serum creatinine; and 6) adverse events.

### 3.3 Convergence assessment and evidence network

**Figure 3:**
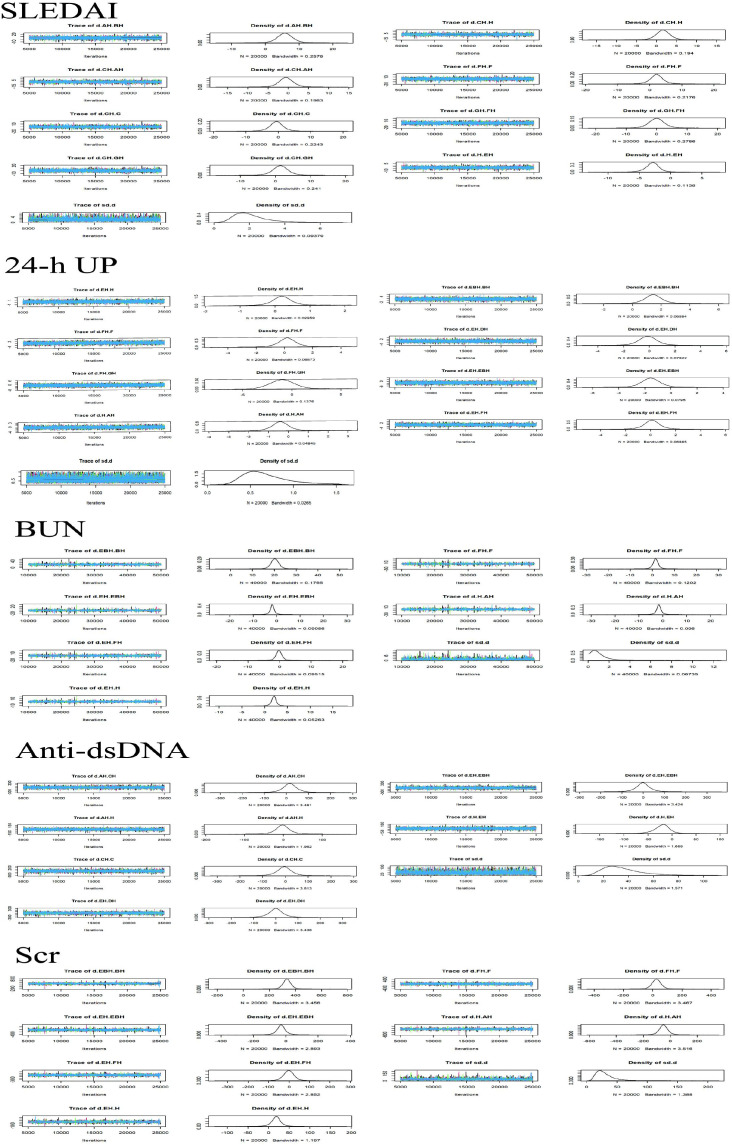
Trace plots and density plots for the Bayesian network meta-analysis models. Trace plots show the sampling history of the model parameters across iterations for each Markov chain, while the density plots display the posterior distributions of the parameters. Visual inspection indicates good mixing and stable convergence of all models. *AH*, PH-350 + conventional pharmacotherapy; *BH*, plasma exchange + conventional pharmacotherapy; *C*, Ig-Therasorb column; *CH*, Ig-Therasorb column + conventional pharmacotherapy; *EH*, DNA280 + conventional pharmacotherapy; *F*, staphylococcal protein A; *FH*, staphylococcal protein A + conventional pharmacotherapy; *GH*, GAM146 column + conventional pharmacotherapy; *H*, conventional pharmacotherapy alone; *EBH*, DNA280 + plasma exchange + conventional pharmacotherapy; *DH*, HA280 + conventional pharmacotherapy.

**Figure 4:**
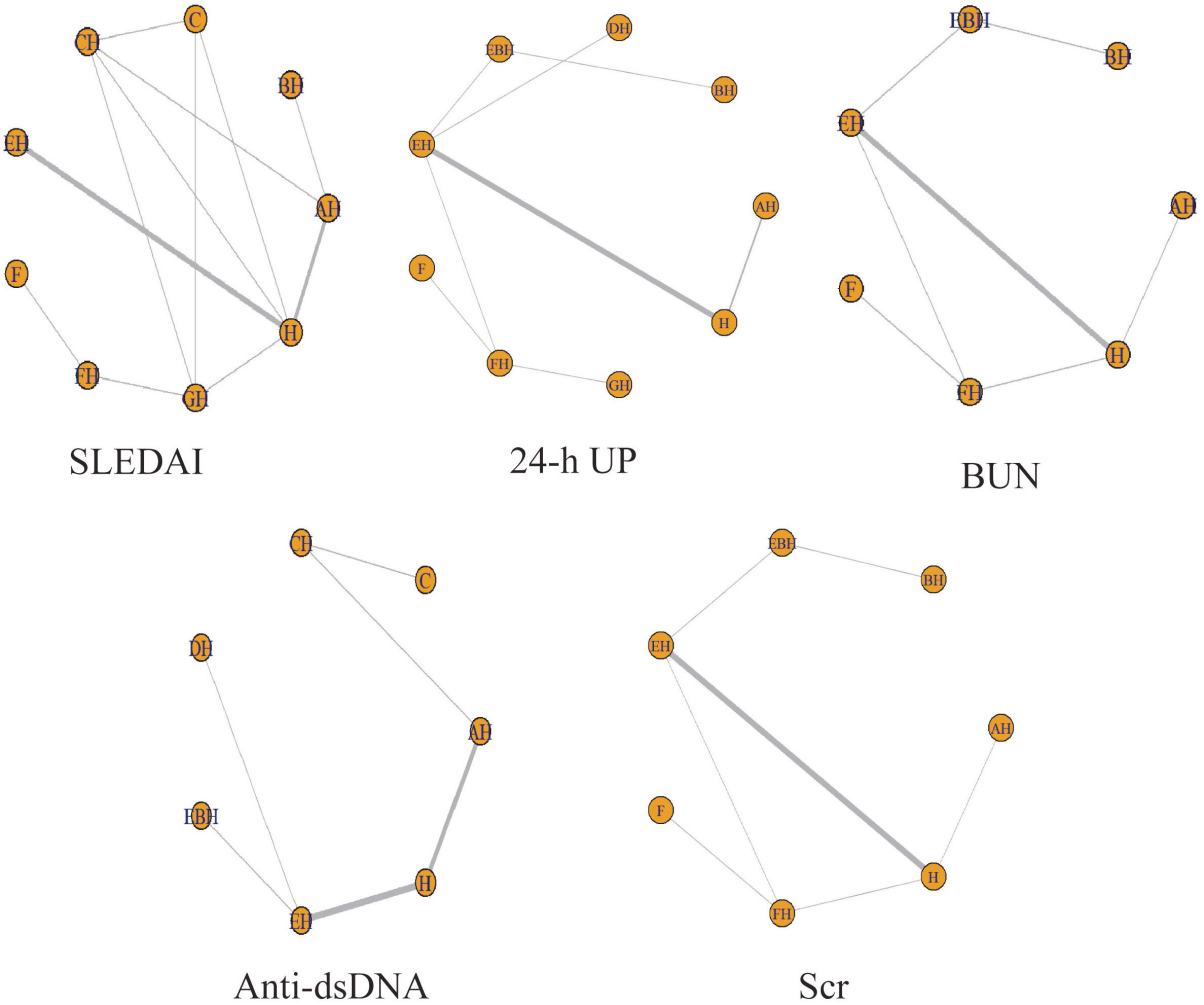
Evidence network for each outcome indicator. *Circles* represent interventions, with the *circle size* proportional to the sample size; *line thickness* reflects the number of studies comparing two interventions; and *lines connecting two nodes* indicate direct head-to-head comparisons between the respective interventions. *AH*, PH-350 + conventional pharmacotherapy; *BH*, plasma exchange + conventional pharmacotherapy; *C*, Ig-Therasorb column; *CH*, Ig-Therasorb column + conventional pharmacotherapy; *EH*, DNA280 + conventional pharmacotherapy; *F*, staphylococcal protein A; *FH*, staphylococcal protein A + conventional pharmacotherapy; *GH*, GAM146 column + conventional pharmacotherapy; *H*, conventional pharmacotherapy alone; *EBH*, DNA280 + plasma exchange + conventional pharmacotherapy; *DH*, HA280 + conventional pharmacotherapy.

The convergence and the stability of the model were assessed using trace plots, density plots, and the PSRF. Specifically, with a maximum of 50,000 iterations, the trace plots demonstrated that majority of the trajectories stabilized, indicating good convergence of the Markov chains after multiple iterations, with estimates tending toward stability and higher reliability of the simulation results. The density plots predominantly exhibited unimodal distributions, signifying a central tendency in the parameter values, with the peak corresponding to the most probable value. All PSRF values were 1.0, indicating satisfactory convergence and stability (see **Figure 3** and **Table 2**). The included studies comprised the following interventions: PH-350 + conventional pharmacotherapy (AH); PE + conventional pharmacotherapy (BH); Ig-Therasorb column (C); Ig-Therasorb column + conventional pharmacotherapy (CH); DNA280 + conventional pharmacotherapy (EH); staphylococcal protein A (F); staphylococcal protein A + conventional pharmacotherapy (FH); GAM146 column + conventional pharmacotherapy (GH); conventional pharmacotherapy alone (H); DNA280 + PE + conventional pharmacotherapy (EBH); and HA280 + conventional pharmacotherapy (DH). **Figure 4** presents the evidence network diagram. Closed loops were formed for the SLEDAI score, BUN, and Scr, thus allowing for inconsistency assessment.

**Table 2:** Potential scale reduction factors (PSRFs) indicating satisfactory convergence of all Bayesian models.

Parameter	Point estimation	Upper 95%Crl
SLEDAI		
d.AH.BH	1.0	1.0
d.CH.AH	1.0	1.0
d.CH.C	1.0	1.0
d.CH.GH	1.0	1.0
d.CH.H	1.0	1.0
d.FH.F	1.0	1.0
d.GH.FH	1.0	1.0
d.H.EH	1.0	1.0
sd.d	1.0	1.0
24-h UP		
d.EBH.BH	1.0	1.0
d.EH.DH	1.0	1.0
d.EH.EBH	1.0	1.0
d.EH.FH	1.0	1.0
d.EH.H	1.0	1.0
d.FH.F	1.0	1.0
d.FH.GH	1.0	1.0
d.H.AH	1.0	1.0
sd.d	1.0	1.0
BUN		
d.EBH.BH	1.0	1.0
d.EH.EBH	1.0	1.0
d.EH.FH	1.0	1.0
d.EH.H	1.0	1.0
d.FH.F	1.0	1.0
d.H.AH	1.0	1.0
sd.d	1.0	1.0
Anti-dsDNA		
d.AH.CH	1.0	1.0
d.AH.H	1.0	1.0
d.CH.C	1.0	1.0
d.EH.DH	1.0	1.0
d.EH.EBH	1.0	1.0
d.H.EH	1.0	1.0
sd.d	1.0	1.0
Scr		
d.EBH.BH	1.0	1.0
d.EH.EBH	1.0	1.0
d.EH.FH	1.0	1.0
d.EH.H	1.0	1.0
d.FH.F	1.0	1.0
d.H.AH	1.0	1.0
sd.d	1.0	1.0

*SLEDAI*, Systemic Lupus Erythematosus Disease Activity Index; *24-h UP*, 24-h urinary protein; *BUN*, blood urea nitrogen; *Scr*, serum creatinine; *AH*, PH-350 + conventional pharmacotherapy; *BH*, plasma exchange + conventional pharmacotherapy; *C*, Ig-Therasorb column; *CH*, Ig-Therasorb column + conventional pharmacotherapy; *EH*, DNA280 + conventional pharmacotherapy; *F*, staphylococcal protein A; *FH*, staphylococcal protein A + conventional pharmacotherapy; *GH*, GAM146 column + conventional pharmacotherapy; *H*, conventional pharmacotherapy alone; *EBH*, DNA280 + plasma exchange + conventional pharmacotherapy; *DH*, HA280 + conventional pharmacotherapy.

### 3.4 Consistency assessment

As shown in **Figure 4**, the network diagrams for SLEDAI, BUN, and Scr all contained closed loops of evidence. Therefore, the node-splitting approach was employed to examine the consistency between the direct and the indirect evidence within these loops.

**Figure 5:**
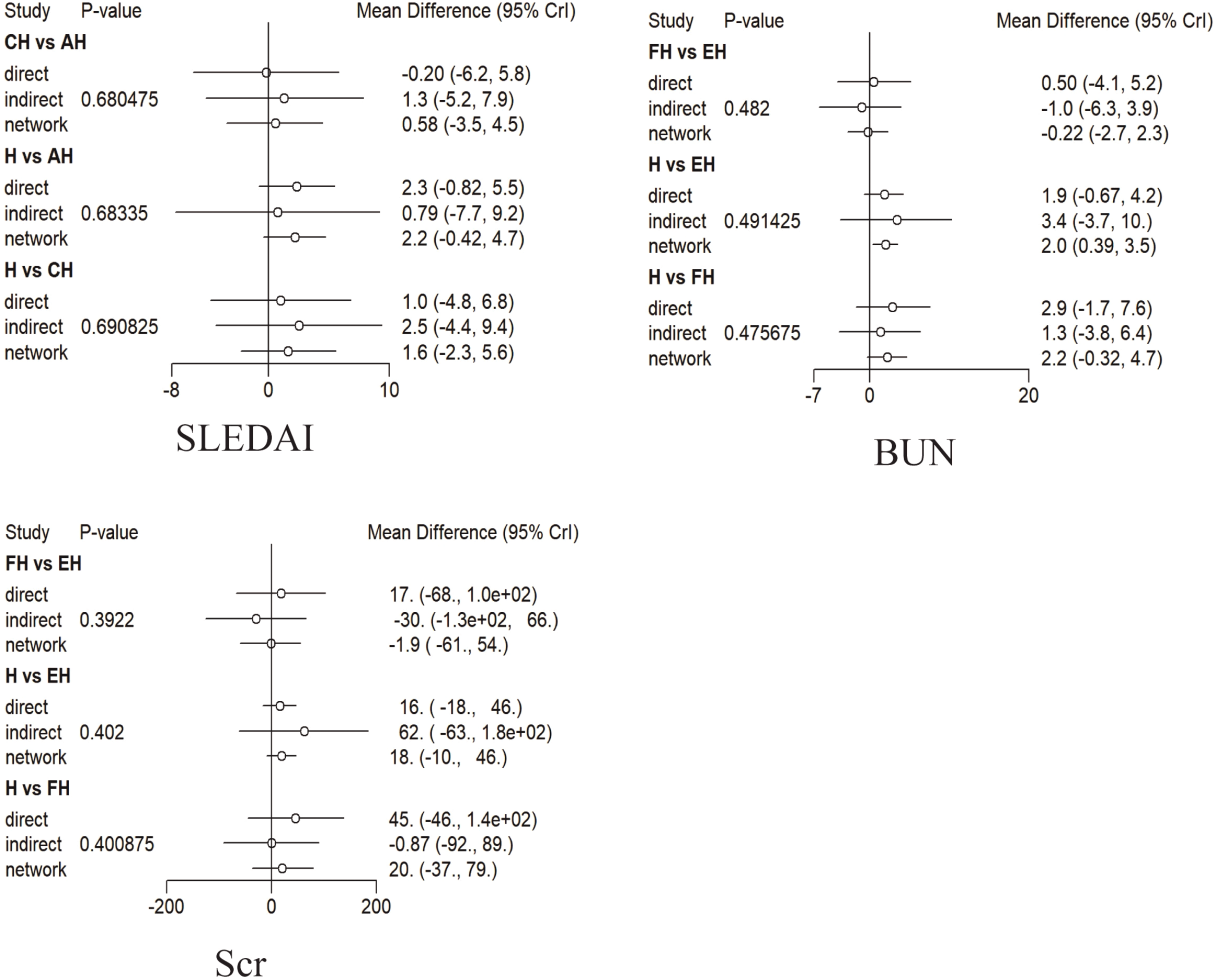
Forest plot of the consistency tests. For each comparison, the *three points* represent the effect estimates derived from direct evidence, indirect evidence, and the network meta-analysis, respectively, and the *horizontal lines* indicate the corresponding 95% credible intervals (CrIs). *AH*, PH-350 + conventional pharmacotherapy; *CH*, Ig-Therasorb column + conventional pharmacotherapy; *EH*, DNA280 immunoadsorption + conventional pharmacotherapy; *FH*, staphylococcal protein A immunoadsorption + conventional pharmacotherapy; *H*, conventional pharmacotherapy alone.

The results of the inconsistency tests are presented in **Figure 5**. For all outcomes, the directions and magnitudes of the effect estimates derived from both direct and indirect comparisons were largely consistent, and no statistically significant inconsistency was detected (all *p*-values >0.05). These findings support the assumption of consistency and justify the use of a consistency model for the primary network meta-analysis. Nevertheless, although no significant inconsistency was identified in the node-splitting analyses, the statistical power of the inconsistency tests may be limited in networks with sparse data. Therefore, the results of the consistency assessment should be interpreted with caution.

### 3.5 Network meta-analysis results

Probability rankings were used to provide an overall comparative framework across interventions. However, these rankings should not be interpreted as evidence of statistically significant superiority, unless supported by corresponding CrIs.

#### 3.5.1 SLEDAI score analysis

**Figure 6:**
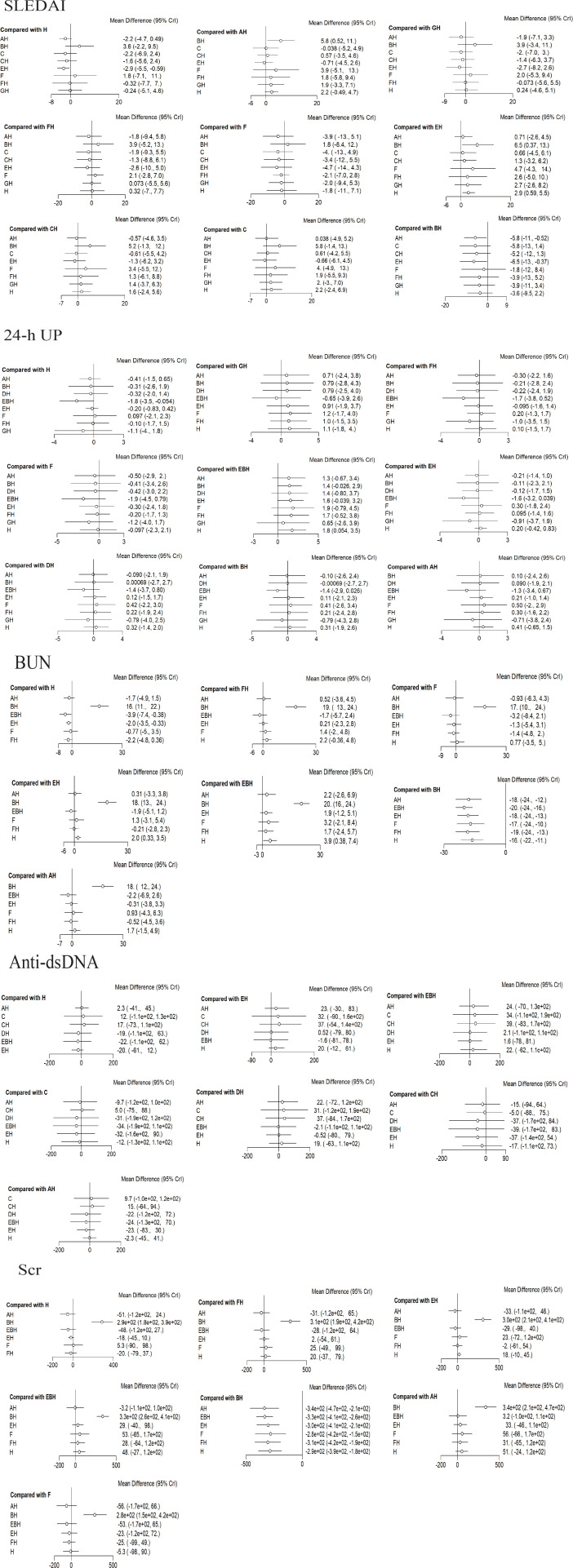
Forest plots of various outcome measures. *Each point* represents the odds ratio (OR) or mean difference (MD) for an intervention, and the *horizontal line* indicates the corresponding 95% credible interval (CrI). *AH*, PH-350 + conventional pharmacotherapy; *BH*, plasma exchange + conventional pharmacotherapy; *C*, Ig-Therasorb column; *CH*, Ig-Therasorb column + conventional pharmacotherapy; *EH*, DNA280 + conventional pharmacotherapy; *F*, staphylococcal protein A; *FH*, staphylococcal protein A + conventional pharmacotherapy; *GH*, GAM146 column + conventional pharmacotherapy; *H*, conventional pharmacotherapy alone; *EBH*, DNA280 + plasma exchange + conventional pharmacotherapy; *DH*, HA280 + conventional pharmacotherapy.

**Figure 7:**
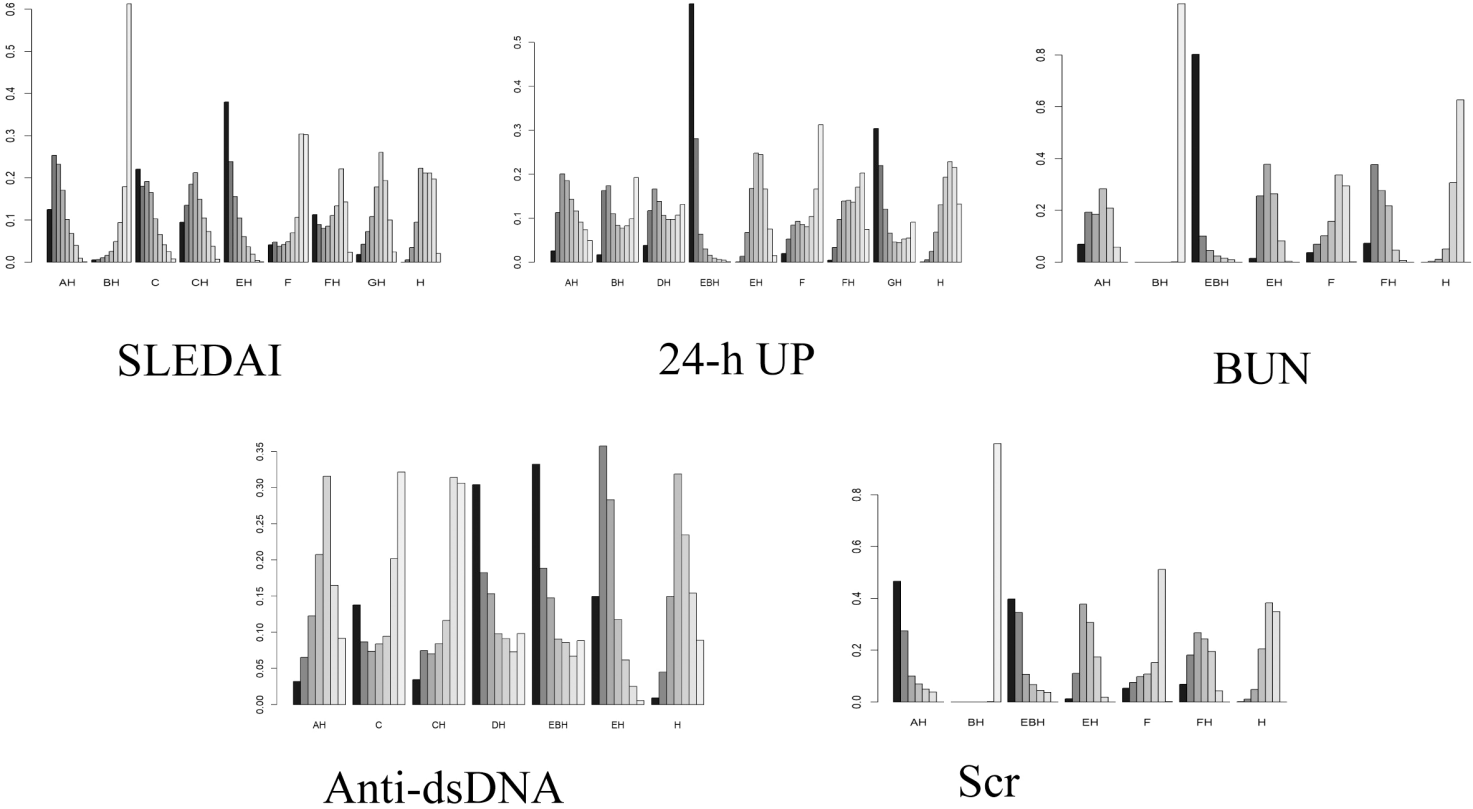
Comparison of the effects of different interventions across outcome indicators. *Letters on the x-axis* represent the different interventions, while the *y*-axis represents the probability that each intervention is ranked as the most effective for the corresponding outcome. The *darkest shading* indicates rank 1, and *taller rank 1* bars reflect a higher probability of being the most effective intervention for that outcome. *AH*, PH-350 + conventional pharmacotherapy; *BH*, plasma exchange + conventional pharmacotherapy; *C*, Ig-Therasorb column; *CH*, Ig-Therasorb column + conventional pharmacotherapy; *EH*, DNA280 + conventional pharmacotherapy; *F*, staphylococcal protein A; *FH*, staphylococcal protein A + conventional pharmacotherapy; *GH*, GAM146 column + conventional pharmacotherapy; *H*, conventional pharmacotherapy alone; *EBH*, DNA280 + plasma exchange + conventional pharmacotherapy; *DH*, HA280 + conventional pharmacotherapy.

There were 12 studies that reported the SLEDAI scores (12–15, 18–20, 22, 24, 27, 28, 31). Analysis showed that, compared with DNA280 immunoadsorption combined with conventional pharmacotherapy, conventional pharmacotherapy alone (MD = 2.9, 95%CrI = 0.59–5.5) and PE combined with conventional pharmacotherapy (MD = 6.5, 95%CrI = 0.37–13) were inferior. PE combined with conventional pharmacotherapy was inferior to PH-350 immunoadsorption combined with conventional pharmacotherapy (MD = 5.8, 95%CrI = 0.52–11). No other comparisons reached statistical significance (see **Figure 6**). The cumulative ranking probability indicated that DNA280 IA combined with conventional pharmacotherapy demonstrated a higher probability of ranking among the top interventions (37.9%), followed by the Ig-Therasorb column (22%), PH-350 + conventional pharmacotherapy (12.4%), staphylococcal protein A + conventional pharmacotherapy (11.3%), Ig-Therasorb column + conventional pharmacotherapy (9.5%), staphylococcal protein A (4.1%), GAM146 column + conventional pharmacotherapy (1.8%), PE + conventional pharmacotherapy (0.5%), and conventional pharmacotherapy alone (0%) (see **Figure 7**; **Table 3**).

**Table 3:** Comprehensive best probability ranking table.

Outcome indicator	AH	BH	C	CH	EH	F	FH	GH	H	EBH	DH
SLEDAI	0.1248125	0.0054875	0.2206250	0.0953375	0.3799875	0.0415125	0.1131250	0.0189000	0.0002875	–	–
24-h UP	0.0264000	0.0168875	–	–	0.0008375	0.0201125	0.0046875	0.3041000	0.0006625	0.5878750	0.0384375
BUN	0.07043125	0.00000000	–	–	0.01465625	0.03766250	0.07377500	–	0.00054375	0.80293125	–
Anti-dsDNA	0.0320375	–	0.1379875	0.0347125	0.1494875	–	–	–	0.0093250	0.3324375	0.3040125
Scr	0.4664250	0.0000125	–	–	0.0127750	0.0533500	0.0685750	–	0.0011000	0.3977625	–

The ranking probabilities reflect the relative comparative tendencies and do not necessarily indicate statistically significant differences between interventions. “–” indicates that the intervention was excluded from this metric. Each value represents the overall probability for the corresponding intervention in this specific metric.

*SLEDAI*, Systemic Lupus Erythematosus Disease Activity Index; *24-h UP*, 24-h urinary protein; *BUN*, blood urea nitrogen; *Scr*, serum creatinine; *AH*, PH-350 + conventional pharmacotherapy; *BH*, plasma exchange + conventional pharmacotherapy; *C*, Ig-Therasorb column; *CH*, Ig-Therasorb column + conventional pharmacotherapy; *EH*, DNA280 + conventional pharmacotherapy; *F*, staphylococcal protein A; *FH*, staphylococcal protein A + conventional pharmacotherapy; *GH*, GAM146 column + conventional pharmacotherapy; *H*, conventional pharmacotherapy alone; *EBH*, DNA280 + plasma exchange + conventional pharmacotherapy; *DH*, HA280 + conventional pharmacotherapy.

#### 3.5.2 24-Hour proteinuria analysis

A total of 14 studies reported 24-h proteinuria (13, 16, 19, 20, 22–24, 27–30, 32, 34, 35). Analysis showed that conventional pharmacotherapy alone was inferior to DNA280 immunoadsorption combined with PE and conventional pharmacotherapy (MD = 1.8, 95%CrI = 0.054–3.5). No other comparisons were statistically significant (see **Figure 6**). The cumulative ranking probability indicated DNA280 that immunoadsorption combined with PE and conventional pharmacotherapy ranked the highest (58.7%), followed by GAM146 column + conventional pharmacotherapy (30.4%), HA280 + conventional pharmacotherapy (3.8%), PH-350 + conventional pharmacotherapy (2.6%), staphylococcal protein A (2%), PE + conventional pharmacotherapy (1.6%), staphylococcal protein A + conventional pharmacotherapy (0.4%), DNA280 + conventional pharmacotherapy (0.08%), and conventional pharmacotherapy alone (0.06%) (see **Figure 7** and **Table 3**).

#### 3.5.3 BUN analysis

There were 10 studies that reported BUN (19, 23, 24, 26, 27, 29, 30, 32, 33, 35). Compared with conventional pharmacotherapy alone, DNA280 immunoadsorption combined with conventional pharmacotherapy (MD = −2, 95%CrI = −3.5 to −0.33) and DNA280 immunoadsorption combined with PE and conventional pharmacotherapy (MD = −3.9, 95%CrI = −7.4 to −0.38) were superior. Compared with PE combined with conventional pharmacotherapy, the following were superior: DNA280 immunoadsorption combined with conventional pharmacotherapy (MD = −18, 95%CrI = −24 to −13), DNA280 immunoadsorption combined with PE and conventional pharmacotherapy (MD = −20, 95%CrI = −24 to −16), PH-350 immunoadsorption combined with conventional pharmacotherapy (MD = −18, 95%CrI = −24 to −12), staphylococcal protein A immunoadsorption (MD = −17, 95%CrI = −24 to −10), conventional pharmacotherapy alone (MD = −16, 95%CrI = −22 to −11), and staphylococcal protein A immunoadsorption combined with conventional pharmacotherapy (MD = −19, 95%CrI = −24 to −13). Other comparisons were not significant (see **Figure 6**). The cumulative ranking probability indicated that DNA280 immunoadsorption combined with PE and conventional pharmacotherapy ranked the highest (80.2%), followed by staphylococcal protein A + conventional pharmacotherapy (7.3%), PH-350 + conventional pharmacotherapy (7%), staphylococcal protein A (3.7%), DNA280 + conventional pharmacotherapy (1.4%), conventional pharmacotherapy alone (0.05%), and PE + conventional pharmacotherapy (0%) (see **Figure 7**; **Table 3**).

#### 3.5.4 Anti-dsDNA antibody analysis

Of the included studies, 13 reported anti-dsDNA antibodies (14–17, 19, 22, 24, 25, 28–31, 33). No comparisons reached statistical significance (see **Figure 6**). The cumulative ranking probability indicated that DNA280 immunoadsorption combined with PE and conventional pharmacotherapy showed the highest ranking probability (33.2%), followed by HA280 + conventional pharmacotherapy (30.4%), DNA280 + conventional pharmacotherapy (14.9%), Ig-Therasorb column (13.7%), Ig-Therasorb column + conventional pharmacotherapy (3.4%), PH-350 + conventional pharmacotherapy (3.2%), and conventional pharmacotherapy alone (0.9%) (see **Figure 7** and **Table 3**).

#### 3.5.5 Serum creatinine analysis

A total of 12 studies reported Scr (19, 20, 23, 24, 26, 27, 29, 30, 32–35). Compared with PE combined with conventional pharmacotherapy, the following were superior: DNA280 immunoadsorption combined with conventional pharmacotherapy (MD = −300, 95%CrI = −410 to −210), DNA280 immunoadsorption combined with PE and conventional pharmacotherapy (MD = −330, 95%CrI = −410 to −260), PH-350 immunoadsorption combined with conventional pharmacotherapy (MD = −340, 95%CrI = −470 to −210), staphylococcal protein A immunoadsorption (MD = −280, 95%CrI = −420 to −150), conventional pharmacotherapy alone (MD = −290, 95%CrI = −390 to −180), and staphylococcal protein A immunoadsorption combined with conventional pharmacotherapy (MD = −310, 95%CrI = −420 to −190). Other comparisons were not significant (see **Figure 6**). The cumulative ranking probability indicated that PH-350 immunoadsorption combined with conventional pharmacotherapy ranked the highest (46.6%), followed by DNA280 immunoadsorption combined with PE and conventional pharmacotherapy (39.7%), staphylococcal protein A + conventional pharmacotherapy (6.8%), staphylococcal protein A (5.3%), DNA280 + conventional pharmacotherapy (1.2%), conventional pharmacotherapy alone (0.1%), and PE + conventional pharmacotherapy (0%) (see **Figure 7**; **Table 3**).

#### 3.5.6 Adverse events

Among the 24 included studies, 17 reported adverse events (**Table 4**). The reported adverse event rates varied across interventions (e.g., 4% for DNA280 + PE + conventional pharmacotherapy and 23% for GAM146 + conventional pharmacotherapy). The most commonly reported events included hypotension, allergic reactions, gastrointestinal symptoms, infections, and bleeding-related events. However, reporting was inconsistent across studies, and several trials provided limited details on event definitions, grading, or follow-up duration. Therefore, these adverse event rates should be interpreted descriptively and should not be considered as comparative safety estimates, and a formal quantitative synthesis of safety outcomes was not feasible.

**Table 4:** Summary of the reported adverse events across the included studies.

Author (year)	Intervention (T/C)		*N* (T/C)		Adverse events (T/C)	
Chee-Yean Loo 2010 (12)	AH	BH	14	14	Thrombocytopenia: 2 cases	Thrombocytopenia: 3 cases
Biesenbach 2009 (13)	FH	GH	6	2	Mild infection: 1 case	Mild infection: 2 cases
Gaubitz 1998 (14)	AH	CH	10	10	Hypotension: 1 case	–
Stummvoll 2004 (15)	CH/C/H/GH		9/7/25/15		Bacterial infection: 3 cases; viral infection: 1 case/–/bacterial infection: 5 cases Viral infection: 3 cases/bacterial infection: 2 cases	
Xu 2015 (16)	DH	EH	31	31	Nausea/vomiting, hypotension: 5 cases	Chills and hypertension: 3 cases
Liang 2017 (17)	EH	H	85	85	Mentioned, but not described	Mentioned, but not described
Chen 2011 (18)	EH	H	30	30	Hypotension: 4 cases; grade I coagulopathy: 2 cases; oozing/hemorrhage: 2 cases	–
Xiao 2015 (19)	EH	H	8	12	Mild rash: 1 case; thrombocytopenia: 1 case; death: 1 case	–
Xie 2024 (21)	EH	H	41	41	Mild gastrointestinal reaction: 1 case; catheter insertion site bleeding: 1 case; catheter-associated venous thrombosis: 1 case	Osteoporosis (complication): 1 case; secondary diabetes: 1 case
Kong 2013 (22)	AH	H	51	55	Allergic reaction: 5 cases; blood pressure decrease: 1 case	–
Xuan 2014 (24)	EH	H	41	41	Allergic reaction: 6 cases	–
Yang 2015 (25)	EH	H	40	40	Chills and fever: 3 cases; hypoglycemia: 3 cases; nausea and vomiting: 4 cases	Chills and fever: 5 cases; hypoglycemia: 4 cases; nausea and vomiting: 5 cases
Yao 2002 (26)	FH	H	15	15	Respiratory tract infection: 2 cases; upper gastrointestinal hemorrhage: 1 case	Respiratory tract infection: 3 cases; upper gastrointestinal hemorrhage: 1 case; thrombocytopenia: 1 case
Kong 2011 (28)	AH	H	39	39	Allergic reaction: 6 cases/blood pressure decrease: 1 case	–
Wang 2019 (29)	EBH	EH	41	41	Nausea and vomiting: 1 case; chills: 1 case	Nausea and vomiting: 4 cases; chills: 2 cases; severe infection: 2 cases
Li 2009 (32)	EH	H	17	19	Nausea with mild blood pressure decrease: 1 case	–
Lai 1999 (33)	AH	H	43	43	Leukopenia: 2 cases; respiratory tract infection: 3 cases; upper gastrointestinal hemorrhage: 1 case; oozing/hemorrhage: 1 case	Leukopenia: 2 cases; respiratory tract infection: 4 cases

“–” indicates that the intervention was excluded from this metric.

*N*, sample size; *T*, trial group; *C*, control group. *AH*, PH-350 + conventional pharmacotherapy; *BH*, plasma exchange + conventional pharmacotherapy; *C*, Ig-Therasorb column; *CH*, Ig-Therasorb column + conventional pharmacotherapy; *EH*, DNA280 + conventional pharmacotherapy; *F*, staphylococcal protein A; *FH*, staphylococcal protein A + conventional pharmacotherapy; *GH*, GAM146 column + conventional pharmacotherapy; *H*, conventional pharmacotherapy alone; *EBH*, DNA280 + plasma exchange + conventional pharmacotherapy; *DH*, HA280 + conventional pharmacotherapy.

### 3.6 Publication bias assessment

**Figure 8:**
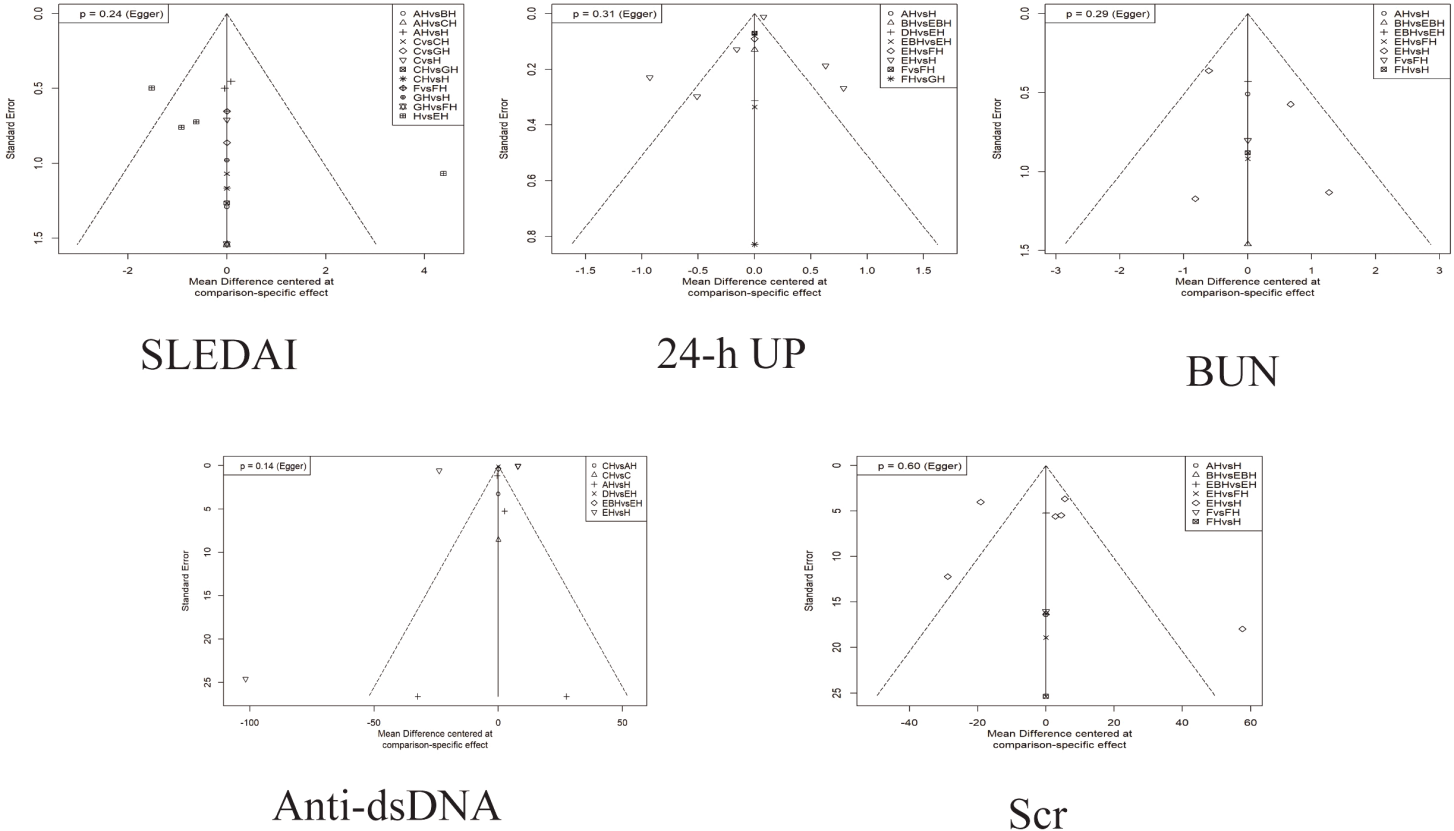
Corrected funnel plots for each outcome indicator. The *x*-axis represents the mean difference (MD) for specific comparisons, reflecting the effect differences between interventions, and the *y*-axis represents the standard error (SE), indicating the precision of the effect estimate, with smaller SE values corresponding to greater precision. *AH*, PH-350 + conventional pharmacotherapy; *BH*, plasma exchange + conventional pharmacotherapy; *C*, Ig-Therasorb column; *CH*, Ig-Therasorb column + conventional pharmacotherapy; *EH*, DNA280 + conventional pharmacotherapy; *F*, Staphylococcal protein A; *FH*, Staphylococcal protein A + conventional pharmacotherapy; *GH*, GAM146 column + conventional pharmacotherapy; *H*, conventional pharmacotherapy alone; *EBH*, DNA280 + plasma exchange + conventional pharmacotherapy; *DH*, HA280 + conventional pharmacotherapy.Tables.

Small-study effects were explored using comparison-adjusted funnel plots and Egger’s test for the SLEDAI scores, 24-h proteinuria, BUN, anti-dsDNA antibodies, and Scr (**Figure 8**). Egger’s tests did not detect statistically significant asymmetry (all *p*-values >0.05). However, given the limited number of studies informing several comparisons and the geographic concentration of the evidence, the ability to detect publication bias or small-study effects may be limited; therefore, these results should be interpreted with caution.

### 3.7 Sensitivity analysis

Across five outcomes (i.e., SLEDAI scores, 24-h proteinuria, BUN, anti-dsDNA antibody levels, and Scr), comparison of the fixed-effects and random-effects models showed that the top-ranked intervention (EBH) remained stable or near stable in terms of the surface under the cumulative ranking curve (SUCRA) rankings. Although some variability was observed among the intermediate-ranked treatments, the overall ranking pattern and the main conclusions were largely consistent between models (**Supplementary material 2**).

## 4 Discussion

To our knowledge, this is the first network meta-analysis in the field of LN to systematically compare the efficacy of six IA columns. Previous research has primarily focused on meta-analyses comparing IA *versus* conventional pharmacotherapy. Given the increasing prevalence of severe LN and the advancements in the targeting capability and safety optimization of IA columns, this study aimed to evaluate the safety and efficacy of six different IA modalities through direct and indirect comparisons. Our study addresses two critical questions: 1) it clarifies the relative advantages and disadvantages of the six IA columns across different disease indicators, and 2) it delineates the differences between these six IA modalities, conventional pharmacotherapy, and PE. These findings can assist healthcare providers in the rational integration of IA therapy with conventional pharmacotherapy when formulating combination regimens for complex clinical scenarios, thereby improving treatment outcomes. This work provides valuable guidance for clinical practice and future decision-making in severe LN while also offering clinical perspectives on the limitations and potential improvements of IA therapy.

The results from the network meta-analysis in this study demonstrated that DNA280 immunoadsorption combined with conventional pharmacotherapy (glucocorticoids + immunosuppressants) showed the highest ranking probability in cumulative probability for optimal efficacy in the SLEDAI assessment. As a core tool for evaluating the disease activity of SLE, SLEDAI integrates 24 clinical and laboratory parameters to comprehensively quantify acute inflammatory activity. Its strengths lie in its standardization, dynamic monitoring capability, and sensitivity to multi-organ involvement, making it widely used for treatment response monitoring and therapeutic decision-making (36). In this network meta-analysis, DNA280 immunoadsorption combined with conventional pharmacotherapy demonstrated a higher probability of ranking among the more effective interventions for reducing disease activity as assessed by SLEDAI. Although DNA280 is designed to selectively bind circulating anti-dsDNA antibodies through antigen–antibody interactions, our analysis did not demonstrate a statistically significant advantage of EH therapy over the other interventions in reducing the anti-dsDNA antibody levels. This discrepancy may reflect inter-study heterogeneity, including differences in the baseline antibody titers, assay methods, treatment duration, and timing of outcome assessment. Therefore, the potential clinical benefits of DNA280-based regimens may not be fully captured by isolated serological endpoints. Mechanistically, selective removal of circulating autoantibodies may provide biological plausibility for improvements in disease activity. However, such plausibility does not necessarily translate into consistent quantitative reductions in circulating anti-dsDNA antibodies across heterogeneous trials (37–40).

However, in the efficacy rankings for anti-dsDNA antibody clearance, 24-h urinary protein quantification, and BUN improvement, the combined regimen of DNA280 immunoadsorption with PE and conventional pharmacotherapy demonstrated optimal clinical benefits. Compared with the SLEDAI results mentioned earlier, this intervention additionally incorporated PE. Anti-dsDNA antibodies, as specific biomarkers for SLE, show serum levels significantly positively correlated with renal pathological activity in LN. The 24-h urinary protein quantification is a core indicator for assessing the severity of renal damage and the prognosis in severe LN, directly reflecting the extent of glomerular filtration barrier disruption caused by immune complex deposition and inflammatory responses. BUN is another critical biochemical marker for the evaluation of renal functional impairment in LN. Elevated levels typically indicate a reduced glomerular filtration rate (GFR) and retention of nitrogenous waste products. The synergistic effect between DNA280 and PE underpins its therapeutic superiority. Importantly, this synergistic effect is likely mediated by complementary mechanisms of selective antibody removal and broad clearance of immune complexes and inflammatory mediators rather than an enhanced anti-dsDNA antibody reduction alone.

In contrast, PE combined with conventional pharmacotherapy showed relatively unfavorable rankings for SLEDAI, BUN, and Scr in the present network meta-analysis. Several clinical and biological factors may have contributed to this observation. PE is a non-selective blood purification technique that removes not only pathogenic autoantibodies and immune complexes but also protective plasma components such as albumin, coagulation factors, complement regulatory proteins, and immunoglobulins. Moreover, PE does not directly suppress autoantibody production and may lead to a post-exchange rebound phenomenon in the absence of adequate immunosuppressive control (41). In addition, patients selected for PE in clinical practice often have more severe or refractory disease, which introduces confounding by indication. Repeated PE procedures may also be associated with hemodynamic instability and fluctuations in renal perfusion, which could adversely affect the short-term BUN and Scr levels. Together, these factors may partly explain the relatively unfavorable performance of PE combined with conventional pharmacotherapy observed in this analysis.

An apparent paradox observed in this network meta-analysis is that the triple therapy that combined DNA280 immunoadsorption, PE, and conventional pharmacotherapy showed favorable efficacy in several direct comparisons, whereas its individual components demonstrated relatively low cumulative ranking probabilities when used alone. This pattern is more consistent with synergistic rather than additive therapeutic effects. In the combined regimen, DNA280 selectively reduces pathogenic anti-dsDNA antibodies, PE facilitates broader clearance of immune complexes and inflammatory mediators, and conventional pharmacotherapy suppresses ongoing autoantibody production. Acting on complementary pathogenic pathways simultaneously may amplify the clinical benefit beyond what can be achieved by any single modality. In contrast, IA or PE alone may be insufficient to counteract continuous immune activation in severe LN. In addition, the ranking probabilities in network meta-analyses may be influenced by limited sample sizes and sparse direct comparisons, which could contribute to unfavorable rankings for single-component therapies. From a translational perspective, the observed benefit of combination therapies is likely driven by their ability to simultaneously reduce the pathogenic autoantibody burden, modulate systemic inflammation, and suppress ongoing immune activation. Further mechanistic details have been reported in experimental studies, but were beyond the scope of this comparative clinical analysis.

From a comparative biological perspective, the differences in the efficacy between IA columns may partly reflect their distinct adsorption targets. DNA280 is designed to selectively remove circulating anti-dsDNA antibodies, which may preferentially translate into improvements in global disease activity indices such as SLEDAI by reducing immune complex formation and downstream inflammatory activation in LN (42). In contrast, PH-350 exhibits broader immunoglobulin G (IgG) adsorption capacity and has been reported in limited studies to adsorb inflammatory mediators such as HMGB-1, a molecule implicated in renal inflammation and tissue injury in LN (43–45). This broader adsorption profile may help explain the relatively favorable tendency of PH-350 combined with conventional pharmacotherapy to improve renal function-related outcomes, including Scr. These mechanistic considerations primarily provide biological plausibility for the observed comparative tendencies rather than definitive causal inference.

From a clinical perspective, IA-based strategies may be more appropriate for patients with severe or refractory LN who exhibit a high autoantibody burden or an inadequate response to conventional pharmacotherapy. However, definitive patient selection criteria require validation in prospective trials. The optimal sequencing of IA, PE, and immunosuppressive therapy could not be determined in this analysis due to the heterogeneous study designs and limited reporting. Future trials specifically designed to address treatment sequencing are warranted. Health economic considerations, including cost-effectiveness and resource utilization, could not be evaluated due to the lack of relevant data in the included studies.

This study compared the efficacy of different IA columns in the treatment of severe LN. While relevant conclusions were drawn, several limitations should be acknowledged, as follows:

i) Limited evidence for specific interventions: Several of the IA columns were supported by only a small number of trials, which may have limited the reliability of the estimated effects. Sparse evidence may also have resulted in imbalanced network geometry, reduced precision of indirect comparisons, and increased uncertainty in ranking probabilities.
ii) Incomplete outcome reporting: Not all clinically important outcomes were consistently reported, limiting the comprehensiveness of the synthesis. Incomplete reporting of the baseline characteristics and outcome data also limited the adjustment for potential confounding and may have influenced the effect estimates.
iii) Clinical and methodological heterogeneity: Substantial heterogeneity existed across studies in terms of treatment protocols, intervention duration, co-interventions, patient characteristics, and outcome assessment methods. Majority of the trials were conducted in Asian populations, with limited representation from Western cohorts, which may have restricted the generalizability. Subgroup analyses (e.g., Asian *versus* Western populations) were not feasible due to the limited data and the uneven geographic distribution.
iv) Methodological quality of the included studies: Majority of the trials are single-center studies conducted in China, with limited reporting of randomization, allocation concealment, and blinding. These deficiencies may have increased the risk of bias, particularly for subjective outcomes. Sensitivity analyses excluding high-risk studies were not feasible as removal of such studies would have substantially reduced the network connectivity and statistical power, yielding unstable estimates. Although the sensitivity analyses using fixed-effects models demonstrated consistent ranking of the leading intervention, some variability among the intermediate treatments was observed, reflecting an underlying heterogeneity. Given the predominance of single-center studies with high risk of bias, the findings should be interpreted with caution.
v) Outcome selection and clinical relevance: The outcomes were primarily surrogate markers (e.g., SLEDAI, proteinuria, BUN, Scr, and anti-dsDNA). Hard clinical endpoints (i.e., ESRD, relapse, mortality, and long-term renal function) and patient-reported outcomes (e.g., quality of life) were rarely reported and could not be analyzed. Thus, the results mainly reflected short-term effects.
vi) Safety analysis limitations: The adverse event definitions, reporting standards, and follow-up durations varied substantially, and several columns were evaluated in only a few studies. Therefore, formal quantitative comparative safety analyses were not feasible, and the adverse event rates should be interpreted descriptively rather than as definitive risk–benefit assessments.
vii) Publication and language bias: A large proportion of the studies were published in Chinese-language journals and conducted in Asian settings, which may have introduced publication and language bias. The funnel plot and Egger’s test results should be interpreted cautiously due to the limited power in sparse networks.
viii) Lack of histopathologic response assessment: None of the included trials consistently reported repeat biopsy findings, ISN/RPS class transitions, or standardized histopathologic response criteria after IA. Therefore, tissue-level renal recovery could not be evaluated. Future trials incorporating repeat biopsy endpoints are warranted.

## 5 Conclusions

Based on this Bayesian network meta-analysis, DNA280-based IA strategies showed relatively favorable probability rankings across multiple outcomes in severe LN, particularly when combined with conventional pharmacotherapy or PE. PH-350 combined with conventional pharmacotherapy showed favorable tendencies for Scr improvement. However, given the heterogeneity and limitations of the available evidence, these findings should be interpreted as hypothesis-generating. Well-designed, multicenter randomized trials with clinically meaningful endpoints are needed.

## Data Availability

The original contributions presented in the study are included in the article/**Supplementary Material**. Further inquiries can be directed to the corresponding author.
