# Supplementary material for: Efficacy and safety of six immunoadsorption treatments for severe lupus nephritis: a Bayesian network meta-analysis and systematic review
Source: Front Immunol. 2026 Mar 13;17:1661291. doi: 10.3389/fimmu.2026.1661291 (PMC13022589; doi:10.3389/fimmu.2026.1661291)
Supplement: Supplementary file 1 [file Table1.docx]

**Supplementary Table 1 PubMed search strategy**

| **Databases:** | **PubMed** |  |  |  |  |  |  |  | |
| --- | --- | --- | --- | --- | --- | --- | --- | --- | --- |
| **Search #** | **Search Query** | | | | | | | | **Search Fields** |
| ****#1**** | ((("Lupus Nephritis"[Mesh]) OR ("Lupus Nephritides"[tiab]) OR ("Lupus Glomerulonephritis"[tiab]) OR ("LN"[tiab])) OR (("Systemic Lupus Erythematosus"[Mesh]) OR ("SLE"[tiab]) OR ("Libman-Sacks Disease"[tiab]))) | | | | | | | | All fields |
| ****#2**** | ((("Immunoadsorption"[Mesh]) OR ("Immunoapheresis"[Mesh]) OR ("Immunoadsorption Therapy"[tiab]) OR ("IA Therapy"[tiab]) OR ("Antibody Adsorption"[tiab])) OR (("Adsorption Columns"[tiab]) OR ("Immunosorbent Columns"[tiab]) OR ("Apheresis Columns"[tiab]))) | | | | | | | | All fields |
| ****#3**** | (("Staphylococcal Protein A"[Mesh]) OR ("Protein A Adsorption"[tiab]) OR ("SPA Adsorption"[tiab]) OR ("Prosorba Column"[tiab])) | | | | | | | | All fields |
| ****#4**** | (("HA280 Column"[tiab]) OR ("HA 280"[tiab]) OR ("HA-280 Immunoadsorption"[tiab]) OR ("HA Type 280"[tiab])) | | | | | | | | All fields |
| ****#5**** | (("DNA Immunoadsorption"[tiab]) OR ("DNA Adsorption Column"[tiab]) OR ("DNA280 Column"[tiab]) OR ("DNA 280"[tiab])) | | | | | | | | All fields |
| ****#6**** | (("PH-350 Column"[tiab]) OR ("PH 350"[tiab]) OR ("PH350 Adsorption"[tiab]) OR ("PH350 Immunosorbent"[tiab])) | | | | | | | | All fields |
| ****#7**** | (("Ig-Therasorb"[tiab]) OR ("Ig Therasorb Column"[tiab]) OR ("IgG Therasorb"[tiab]) OR ("Therasorb IA"[tiab])) | | | | | | | | All fields |
| ****#8**** | (("GAM146 Column"[tiab]) OR ("GAM 146"[tiab]) OR ("GAM146 Immunoadsorbent"[tiab]) OR ("Globaffin Column"[tiab])) | | | | | | | | All fields |
| ****#9**** | (("Immunosorba"[tiab]) OR ("Ig Adsopak"[tiab]) OR ("Excorim Column"[tiab]) OR ("Miro Adsorber"[tiab]) OR ("Globaffin"[tiab])) | | | | | | | | All fields |
| ****#10**** | #2 OR #3 OR #4 OR #5 OR #6 OR #7 OR #8 OR #9 | | | | | | | | All fields |
| ****#11**** | (("Randomized Controlled Trial"[pt]) OR ("Controlled Clinical Trial"[pt]) OR ("Randomized"[tiab]) OR ("Randomly"[tiab]) OR ("Placebo"[tiab]) OR ("Clinical Trial"[pt])) NOT ("mankind"[Mesh] NOT "Humans"[Mesh])) | | | | | | | | All fields |
| ****#12**** | #1 AND #10 AND #11 | | | | | | | | All fields |
