# Supplementary material for: Efficacy and safety of six immunoadsorption treatments for severe lupus nephritis: a Bayesian network meta-analysis and systematic review
Source: Front Immunol. 2026 Mar 13;17:1661291. doi: 10.3389/fimmu.2026.1661291 (PMC13022589; doi:10.3389/fimmu.2026.1661291)
Supplement: Supplementary file 2 [file Table2.docx]

1 SLEDAI SCORE

> model<-mtc.model(network,type="consistency",n.chain=4,likelihood="normal",link="identity",linearModel="fixed")

> results <- mtc.run(model, n.adapt = 20000, n.iter = 50000, thin = 1)

Compiling model graph

Resolving undeclared variables

Allocating nodes

Graph information:

Observed stochastic nodes: 26

Unobserved stochastic nodes: 20

Total graph size: 372

Initializing model

|**************************************************| 100%

> summary(results)

Results on the Mean Difference scale

Iterations = 1:50000

Thinning interval = 1

Number of chains = 4

Sample size per chain = 50000

1. Empirical mean and standard deviation for each variable,

plus standard error of the mean:

Mean SD Naive SE Time-series SE

d.AH.BH 5.8019 1.2840 0.0028711 0.003036

d.CH.AH -0.7575 0.9530 0.0021310 0.014672

d.CH.C -0.6049 0.9394 0.0021007 0.011422

d.CH.GH 1.3954 1.1601 0.0025942 0.011523

d.CH.H 1.5336 0.9407 0.0021035 0.014674

d.FH.F 2.1004 0.6530 0.0014602 0.002276

d.GH.FH -0.1005 1.5410 0.0034458 0.004025

d.H.EH -2.2292 0.3424 0.0007656 0.001264

2. Quantiles for each variable:

2.5% 25% 50% 75% 97.5%

d.AH.BH 3.2759 4.9371 5.80201 6.67101 8.313

d.CH.AH -2.6285 -1.4016 -0.75895 -0.11627 1.122

d.CH.C -2.4377 -1.2405 -0.60805 0.02638 1.246

d.CH.GH -0.8706 0.6116 1.39490 2.18071 3.658

d.CH.H -0.3167 0.8998 1.53158 2.16613 3.381

d.FH.F 0.8214 1.6586 2.10170 2.53988 3.382

d.GH.FH -3.1181 -1.1386 -0.09768 0.93414 2.924

d.H.EH -2.8993 -2.4606 -2.22993 -1.99898 -1.559

-- Model fit (residual deviance):

Dbar pD DIC

45.99826 20.01754 66.01581

26 data points, ratio 1.769, I^2 = 46%

> sucra(ranks)

AH BH C CH EH F FH GH H

0.1547350 0.9618963 0.1946256 0.3441112 0.1788312 0.8569281 0.5382675 0.6171206 0.6534844

2 24-hour proteinuria.

> model<-mtc.model(network,type="consistency",n.chain=4,likelihood="normal",link="identity",linearModel="fixed")

> results <- mtc.run(model, n.adapt = 20000, n.iter = 50000, thin = 1)

Compiling model graph

Resolving undeclared variables

Allocating nodes

Graph information:

Observed stochastic nodes: 28

Unobserved stochastic nodes: 22

Total graph size: 400

Initializing model

|**************************************************| 100%

> summary(results)

Results on the Mean Difference scale

Iterations = 1:50000

Thinning interval = 1

Number of chains = 4

Sample size per chain = 50000

1. Empirical mean and standard deviation for each variable,

plus standard error of the mean:

Mean SD Naive SE Time-series SE

d.EBH.BH 1.43894 0.11655 2.606e-04 1.122e-03

d.EH.DH -0.12397 0.31312 7.002e-04 1.312e-03

d.EH.EBH -1.55894 0.32738 7.320e-04 1.534e-03

d.EH.FH 0.09985 0.09130 2.042e-04 2.984e-04

d.EH.H 0.11200 0.01181 2.641e-05 3.566e-05

d.FH.F 0.19992 0.07074 1.582e-04 4.281e-04

d.FH.GH -1.00330 1.03527 2.315e-03 4.755e-03

d.H.AH -0.41010 0.04993 1.116e-04 1.794e-04

2. Quantiles for each variable:

2.5% 25% 50% 75% 97.5%

d.EBH.BH 1.21721 1.36300 1.4396 1.51553 1.6624

d.EH.DH -0.73756 -0.33552 -0.1228 0.08721 0.4896

d.EH.EBH -2.19947 -1.78066 -1.5579 -1.33827 -0.9160

d.EH.FH -0.07949 0.03826 0.1002 0.16162 0.2782

d.EH.H 0.08879 0.10409 0.1120 0.11998 0.1351

d.FH.F 0.06082 0.15229 0.1999 0.24780 0.3383

d.FH.GH -3.03624 -1.70314 -1.0032 -0.30201 1.0149

d.H.AH -0.50780 -0.44380 -0.4101 -0.37653 -0.3123

-- Model fit (residual deviance):

Dbar pD DIC

64.27909 22.04638 86.32547

28 data points, ratio 2.296, I^2 = 58%

> sucra(ranks)

AH BH DH EBH EH F FH GH H

0.7104244 0.5034931 0.5142469 0.9656794 0.4701031 0.0512950 0.2962250 0.7339037 0.2546294

3 BUN.

> model<-mtc.model(network,type="consistency",n.chain=4,likelihood="normal",link="identity",linearModel="fixed")

> results <- mtc.run(model, n.adapt = 20000, n.iter = 50000, thin = 1)

Compiling model graph

Resolving undeclared variables

Allocating nodes

Graph information:

Observed stochastic nodes: 20

Unobserved stochastic nodes: 16

Total graph size: 280

Initializing model

|**************************************************| 100%

> summary(results)

Results on the Mean Difference scale

Iterations = 1:50000

Thinning interval = 1

Number of chains = 4

Sample size per chain = 50000

1. Empirical mean and standard deviation for each variable,

plus standard error of the mean:

Mean SD Naive SE Time-series SE

d.EBH.BH 20.2600 1.4792 0.0033077 0.008351

d.EH.EBH -1.9185 0.4315 0.0009650 0.002430

d.EH.FH -0.1748 0.6540 0.0014624 0.002695

d.EH.H 2.1167 0.2799 0.0006259 0.001036

d.FH.F 1.4377 0.8032 0.0017959 0.003440

d.H.AH -1.7041 0.5084 0.0011368 0.001944

2. Quantiles for each variable:

2.5% 25% 50% 75% 97.5%

d.EBH.BH 17.3844 19.2729 20.2576 21.2502 23.1400

d.EH.EBH -2.7651 -2.2091 -1.9200 -1.6279 -1.0716

d.EH.FH -1.4583 -0.6150 -0.1749 0.2662 1.1065

d.EH.H 1.5698 1.9277 2.1161 2.3062 2.6630

d.FH.F -0.1368 0.8974 1.4376 1.9795 3.0117

d.H.AH -2.7015 -2.0466 -1.7051 -1.3602 -0.7073

-- Model fit (residual deviance):

Dbar pD DIC

22.64288 16.02321 38.66608

20 data points, ratio 1.132, I^2 = 16%

> sucra(ranks)

AH BH EBH EH F FH H

5.411000e-01 3.416667e-05 9.972833e-01 6.742958e-01 3.626275e-01 7.240492e-01 2.006100e-01

4 anti-dsDNA antibody levels

> modelume<-mtc.model(network,type="ume",n.chain=4,likelihood="normal",link="identity",linearModel="fixed")

> resultsume <- mtc.run(modelume, n.adapt = 20000, n.iter = 50000, thin = 1)

Compiling model graph

Resolving undeclared variables

Allocating nodes

Graph information:

Observed stochastic nodes: 26

Unobserved stochastic nodes: 19

Total graph size: 294

Initializing model

|**************************************************| 100%

> summary(resultsume)

Results on the Mean Difference scale

Iterations = 1:50000

Thinning interval = 1

Number of chains = 4

Sample size per chain = 50000

1. Empirical mean and standard deviation for each variable,

plus standard error of the mean:

Mean SD Naive SE Time-series SE

d.AH.CH 15.0215 3.2725 0.0073175 0.0111771

d.AH.H -2.1336 1.1510 0.0025737 0.0041077

d.C.CH 4.9441 8.5767 0.0191781 0.0546400

d.DH.EH -0.4203 0.1261 0.0002820 0.0005594

d.EBH.EH 1.5999 0.3612 0.0008077 0.0012950

d.EH.H 0.4238 0.1520 0.0003398 0.0008477

2. Quantiles for each variable:

2.5% 25% 50% 75% 97.5%

d.AH.CH 8.6137 12.8135 15.0228 17.2299 21.4382

d.AH.H -4.3897 -2.9106 -2.1331 -1.3555 0.1177

d.C.CH -11.8163 -0.8471 4.9280 10.7336 21.7566

d.DH.EH -0.6668 -0.5055 -0.4203 -0.3354 -0.1737

d.EBH.EH 0.8933 1.3560 1.6004 1.8432 2.3092

d.EH.H 0.3476 0.3968 0.4227 0.4486 0.4978

-- Model fit (residual deviance):

Dbar pD DIC

2883.1879 39.2549 2922.4429

26 data points, ratio 110.9, I^2 = 99%

> sucra(ranks)

AH C CH DH EBH EH H

0.32324250 0.19934333 0.04673917 0.56368417 0.98943167 0.81659917 0.56096000

5 serum creatinine.

> modelume<-mtc.model(network,type="ume",n.chain=4,likelihood="normal",link="identity",linearModel="fixed")

> resultsume <- mtc.run(modelume, n.adapt = 20000, n.iter = 50000, thin = 1)

Compiling model graph

Resolving undeclared variables

Allocating nodes

Graph information:

Observed stochastic nodes: 24

Unobserved stochastic nodes: 19

Total graph size: 277

Initializing model

|**************************************************| 100%

> summary(resultsume)

Results on the Mean Difference scale

Iterations = 1:50000

Thinning interval = 1

Number of chains = 4

Sample size per chain = 50000

1. Empirical mean and standard deviation for each variable,

plus standard error of the mean:

Mean SD Naive SE Time-series SE

d.AH.H 50.92 16.408 0.036689 0.06669

d.BH.EBH -334.89 16.268 0.036377 0.66515

d.EBH.EH 29.58 5.206 0.011641 0.01782

d.EH.FH 17.15 18.902 0.042266 0.06488

d.EH.H 19.86 2.173 0.004858 0.01308

d.F.FH -25.17 16.046 0.035880 0.07736

d.FH.H 45.10 25.327 0.056633 0.09611

2. Quantiles for each variable:

2.5% 25% 50% 75% 97.5%

d.AH.H 18.657 39.868 50.96 62.01 83.075

d.BH.EBH -367.141 -345.557 -334.98 -323.91 -303.644

d.EBH.EH 19.329 26.070 29.59 33.08 39.789

d.EH.FH -19.855 4.389 17.12 29.96 54.078

d.EH.H 15.601 18.405 19.86 21.33 24.131

d.F.FH -56.614 -35.977 -25.20 -14.34 6.335

d.FH.H -4.732 28.055 45.14 62.12 94.733

-- Model fit (residual deviance):

Dbar pD DIC

59.35307 19.02381 78.37688

24 data points, ratio 2.473, I^2 = 61%

> sucra(ranks)

AH BH EBH EH F FH H

0.9003875 0.0024375 0.9064008 0.5779808 0.2617808 0.5610050 0.2900075
